# Myopia development: multifactorial interplay, molecular mechanisms and possible strategies

**DOI:** 10.3389/fmed.2025.1638184

**Published:** 2025-08-26

**Authors:** Lihong Huang, Dazheng Zhang, Jing Zhou

**Affiliations:** ^1^Dujiangyan Medical Centre, Chengdu, China; ^2^Chengdu University of Traditional Chinese Medicine, Chengdu, China; ^3^Dujiangyan Juvenile Myopia Prevention and Treatment Center, Chengdu, China

**Keywords:** myopia, neurotransmitters, hormones, intracellular signaling pathways, interventions

## Abstract

Myopia is a global visual impairment, and its pathological mechanism involves the complex multifactorial interplay of neurotransmitters, hormones and intracellular signaling pathways. Dopamine inhibits axial growth by activating D2 receptor-CAMP pathway, while GABA energy signals maintain refractive stability by regulating retinal excitation-inhibition balance. Sex hormones and vitamin D play a dual role by regulating scleral ECM metabolism, the former enhances corneal strength and may promote axial elongation during puberty, while the latter exhibits dose-dependent biphasic effects on collagen synthesis via VDR signaling. In the core signaling pathway, the hypoxia-HIF-1α-MMP-2 axis drives sclera ECM degradation, while TGF-β and Wnt/β-catenin pathways synergically regulate fibroblast proliferation and migration. In response to these mechanisms, multi-target intervention strategies show potential: low-dose atropine combined with outdoor light can synergically inhibit axial growth. However, while emerging technologies like gene editing (e.g., CRISPR targeting HIF-1α) and targeted MMP-2 inhibitors are entering preclinical validation, their clinical translation faces substantial hurdles. For CRISPR-based approaches, critical challenges include mitigating off-target editing risks and developing safe, efficient *in vivo* delivery systems to the relevant ocular tissues. Future studies need to integrate molecular mechanisms and interdisciplinary approaches to rigorously evaluate efficacy, safety, and feasibility in order to develop precise prevention and control programs to cope with the epidemic trend of myopia.

## 1 Introduction

Signal pathways refer to a series of interconnected biomolecules and chemical reactions that transmit specific signals within or between cells, thereby regulating physiological functions and behaviors (1). By 2050, nearly 50% of the global population is projected to be affected by myopia (2). In the eye, three key structures—the retina, choroid, and sclera—interact dynamically to mediate refractive development. The retina is a light-sensitive sensory layer located on the posterior inner surface of the eye (3). The retinal pigment epithelium, a layer of highly specialized pigment cells between the neural retina and the vascular choroid, forms a barrier that maintains retinal homeostasis (4). The choroid, the vascular layer of the eye, supplies oxygen and nutrients to the outer retina and plays an active role in orthokeratology by mechanically adjusting its thickness in response to optic nerve defocus, thereby aligning the retina with the eye’s focal plane (5). The sclera (6), a fibrous outer layer, undergoes pathological thinning during myopia due to extracellular matrix (ECM) degradation, driven by aberrant signaling cascades (7).

Myopia development is governed by a complex interplay of neurotransmitters, hormones, and intracellular signaling pathways (8). Dopamine inhibits axial elongation through D2 receptor-cAMP pathways, with light exposure amplifying its protective role, while GABA and glutamate maintain retinal excitatory-inhibitory balance, disruptions of which correlate with scleral remodeling (9–11). Melatonin and serotonin link circadian rhythms to ocular growth patterns, and hormonal crosstalk—such as insulin/glucagon-mediated ECM metabolism and estrogen’s dual roles in corneal integrity and axial elongation—further modulates eye development (12–14). Vitamin D counteracts MMP-2-driven ECM degradation via VDR signaling, highlighting the integrated regulatory network (15). Hypoxia-driven pathways play a central role in scleral thinning: reduced choroidal perfusion stabilizes HIF-1α, upregulating MMP-2 to degrade collagen, a process exacerbated by inflammatory signals like TNF-α/NF-κB. Concurrently, TGF-β activates Smad and non-Smad pathways (e.g., PI3K/AKT, MAPK/ERK) to regulate collagen synthesis and fibroblast differentiation, while Wnt/β-catenin promotes fibroblast migration, mitigated by DKK-1 inhibition (16–18). Prostaglandins and tyrosine metabolism-related genes foster a pro-myopic microenvironment by altering vascular permeability and cytokine release, with retinoic acid (RA) modulating TGF-β2 secretion to influence scleral stiffness.

Critical research gaps persist, including unresolved crosstalk between MAPK/ERK and PI3K/AKT pathways in fibroblast differentiation, and estrogen’s paradoxical effects on corneal strength versus axial elongation. Clinically, the long-term safety of dopamine agonists and low-dose atropine remains uncertain, while CRISPR-based HIF-1α knockout requires preclinical validation. Combinatorial strategies—such as pairing blue light exposure with MMP-2 inhibitors—and personalized interventions targeting genetic variants (e.g., TGF-β codon 10) offer promising avenues (19). Bridging molecular insights (e.g., hypoxia-HIF-1α-MMP-2 axis) with innovations like gene editing is essential to curb myopia progression, necessitating prioritized research on multiple pathways interaction and translational trials to advance precision ocular therapies.

## 2 Neurotransmitters

Retinal neurotransmitter networks drive myopia through sequential biomechanical pathways: (1) High-intensity light stimulates DA release, which activates scleral fibroblast D2 receptors to inhibit ERK signaling, thereby suppressing ECM degradation and restraining ocular expansion. (2) Circadian disruption suppresses melatonin and DA synthesis, while acetylcholine (ACh) potentiates growth via M4 receptor-mediated antagonism of D1-like DA signaling and M2/M4-driven cAMP inhibition in scleral fibroblasts. (3) Reducing DA and GABAergic tone dysregulates retinal inhibition, exacerbating glutamate excitotoxicity—triggering retinal ganglion cell apoptosis and impairing chloride transport to increase intravitreal fluid volume. (4) These perturbations converge with inflammatory mediators and disrupted tyrosine metabolism to upregulate MMPs via RARα/NF-κB pathways, directly degrading scleral ECM. (5) Serotonin further modulates this cascade through 5-HT2A receptor-mediated growth potentiation under deprivation. Ultimately, synaptic imbalances and inflammatory signaling induce scleral thinning and biomechanical failure, constituting the final common pathway for axial elongation (Table 1).

**Table 1:** Neurotransmitters in myopia: mechanisms and therapeutic implications.

Neurotransmitter	Function and mechanism	Related pathways	Genetic factors	Treatment strategies
Dopamine	Inhibits axial elongation; light-dependent release via D2 receptor-ERK axis; regulates circadian rhythms	D2 receptor-cAMP pathway, ERK pathway	Genetic variants	Dopamine agonists (e.g., apomorphine 0.1%); Light therapy (≥1,000 lux, 480 nm dominant)
5-HT (serotonin)	Modulates retinal function/structure; 5-HT2A receptor overexpression linked to myopia; influences norepinephrine/epinephrine levels	5-HT receptors (e.g., 5-HT2A)	Increased 5-HT2A expression in deprived guinea pig models	5-HT receptor modulators
GABA	Inhibitory neurotransmitter; maintains retinal excitation-inhibition balance; regulates K^+^/Ca^2+^ channels to suppress abnormal axial growth	GABAA, GABAB, GABAC	Suppressed GABAergic signaling in form-deprivation myopia models	GABA receptor antagonists
Glutamate	Primary excitatory neurotransmitter; mediates phototransduction; excess leads to excitotoxicity and retinal structural changes	Glutamate receptors	Elevated glutamate levels in myopic eyes	Modulating glutamatergic balance; antioxidants to reduce excitotoxicity
Acetylcholine (ACh)	Regulates phototransduction (M1), inhibits cAMP (M2/M4), antagonizes dopamine (M4), modulates vasculature (M3)	M1, M2/M4, and cAMP	High M3 receptor expression in ciliary muscle	Non-selective antagonists subtype-selective targeting
Melatonin	Regulates circadian rhythms; suppresses dopamine release; nocturnal rise indirectly influences axial growth	Melatonin receptors (MT1/MT2)	Artificial light at night disrupts rhythms, reducing melatonin levels	Controlling nighttime light exposure; melatonin analogs for rhythm regulation

### 2.1 Dopamine

Dopamine, a critical retinal neurotransmitter, regulates ocular growth and serves as a protective factor against myopia by inhibiting axial elongation (20).

Light exposure critically influences dopamine dynamics (21), with observational studies correlating high-intensity outdoor light with reduced myopia incidence (22). Experimental evidence demonstrates that specific wavelengths directly modulate ocular growth: Causal studies in animal models confirm that blue/violet light inhibits axial elongation by stimulating retinal dopamine release, while red light promotes excessive growth (23, 24). Far/near-infrared (FR/NIR) light may indirectly stabilize dopamine levels via nitric oxide (NO)-mediated choroidal blood flow enhancement, alleviating scleral hypoxia (25–27).

Causal studies in animal models confirm that blue/violet light (460–480 nm) inhibits axial elongation by stimulating retinal dopamine release, while red light (>600 nm) promotes excessive growth through concurrent dopamine suppression and melatonin disinhibition (28–31). Crucially, wavelength-dependent melatonin regulation mediates this duality: (1) Blue light suppresses retinal melatonin synthesis via melanopsin-dependent ipRGC signaling, amplifying dopamine’s anti-myopic effects. (2) Red light fails to activate melanopsin, permitting elevated melatonin which antagonizes dopamine signaling and promotes axial elongation. Clinical validation comes from children wearing blue-blocking lenses (32), which reduced melatonin suppression and accelerated myopia progression by 1.5 mm versus controls.

Mechanistically, dopamine inhibits axial elongation primarily through D2 receptor activation of the ERK pathway in scleral fibroblasts, as evidenced by: Pharmacological interventions (33): DA agonists suppress eye growth, while antagonists exacerbate it in controlled studies. Genetic support: Variants in ERK pathway genes (e.g., RASGRF1 rs6495367, PTPN5 rs1550870) correlate with myopia susceptibility in Asian cohorts, though direct causal links require functional validation (34–36).

Daytime light synchronizes dopamine release with circadian rhythms to regulate eye growth. Conversely, artificial nighttime light disrupts circadian rhythms, suppressing melatonin and impairing dopamine regulation, epidemiological studies associate this with increased myopia risk (37, 38).

However, recent studies challenge dopamine as a strict requirement for myopia control: Atropine retains anti-myopic efficacy in animal models without measurable increases in retinal dopamine, suggesting alternative or parallel pathways (39). Atropine’s mechanism remains incompletely resolved, with proposed actions ranging from direct retinal modulation to scleral remodeling (40).

### 2.2 5-HT

Serotonin [5-hydroxytryptamine (5-HT)] is a neurotransmitter synthesized in the central nervous system and widely distributed in the peripheral and central nervous systems (41). In guinea pig models of lens-induced myopia, significantly higher levels of 5-HT and 5-HT2A receptors have been observed. Exposure to flashing light and form deprivation further increases the expression of the 5-HT2A receptor while decreasing concentrations of norepinephrine and epinephrine in their eyes (42, 43). Photoreceptors primarily use glutamate/aspartate as neurotransmitters, while interneurons utilize GABA, glycine, acetylcholine, and dopamine, with bipolar cells also using glutamate (44). Despite this, 5-HT plays a neuromodulator role in the retina. Anaplastic cells, a type of interneuron, are capable of synthesizing and releasing 5-HT, acting as serotonergic neurons (45). These cells form synapses with bipolar and ganglion cells, which express various types of 5-HT receptors (5-HTR) mediating the neuromodulator effects of 5-HT. Thus, 5-HT in the retina, although not the primary neurotransmitter for photoreceptors, has a significant modulatory influence on retinal function and structure, impacting eye growth and potentially myopia development (46). Serotonin receptors in the retina influence signaling pathways contributing to eye development and refractive changes (47). Research shows altered serotonin levels in myopic eyes, suggesting serotonin’s involvement in myopia progression (48, 49). Modulating serotonin receptors could offer therapeutic avenues for controlling myopia, highlighting serotonin’s role in retinal health and refractive stability.

### 2.3 GABA

Gamma-aminobutyric acid (GABA) is an essential inhibitory neurotransmitter in the retina, maintaining the balance of excitatory and inhibitory signals within retinal circuitry (50). GABAergic signaling regulates the opening of potassium and calcium channels in retinal cells, impacting signal transmission and eye development (51). The inhibition of myopic eye growth is linked to GABA’s inhibitory effects, with disruptions in GABA signaling leading to changes in eye growth and myopia progression. There are three distinct families of GABA receptors: GABAA, GABAB, and GABAC. GABAA receptors are ligand-gated ion channels, GABAB receptors are G-protein-coupled receptors, and GABAC receptors are transmitter-gated chloride channels (52). Activation of GABAB receptors, which interact with neuronal inward, rectifier potassium, and voltage-gated calcium channels, mediates slow synaptic depression (53, 54). GABA signaling in the retina, which transitions from excitatory to inhibitory, plays a crucial role in retinal development and function (55). Disruptions in this GABAergic regulation could impact retinal processing and potentially contribute to myopia development (56). Patterns of retinal GABA changes and increased levels of GAD, GABA, and mRNA in the visual cortex suggest that GABA’s roles in the retinal and suprachiasmatic pathways are interrelated (57). Studies have shown that GABA antagonists can prevent myopia development by utilizing the scleral mechanism of the RPE/choroid, highlighting the significance of GABAergic signaling in myopia management (58, 59).

The GABA signaling pathway also plays a crucial role in myopia by regulating potassium and calcium channels within retinal cells, essential for controlling retinal signal transmission and overall eye development (60). Induction of form-deprivation myopia (FDM) leads to significant inhibition of the retinal ligand-gated chloride channel transporter pathway via inhibition of the glycine, GABAA, and GABA ionotropic receptors, reducing fluid flow through the retina to the choroid (7). This inhibition leads to a rapid increase in intravitreal fluid and lengthening of the ocular axis. In FDM, the multiple pathways interaction between dopamine and GABAergic neurotransmitter pathways is critical, with exposure to fluorescent light reducing GABA inhibitory activity in deprived eyes (61). The roles of GABA receptors in mediating feedback between retinal cells and regulating intracellular messengers underscore their importance in eye growth and refractive development. Understanding these mechanisms can help develop targeted therapies to manage myopia progression effectively.

### 2.4 Glutamate and tyrosine

Glutamate is the primary excitatory neurotransmitter in the retina, crucial for transmitting visual signals from photoreceptors to bipolar and ganglion cells. It plays a fundamental role in visual processing and eye growth regulation. Specifically, glutamate excitotoxicity has been implicated in inducing retinal ganglion cell apoptosis, neuronal degeneration within the inner nuclear layer (particularly bipolar and amacrine cells), and potential damage to photoreceptor integrity (62, 63). These structural alterations are associated with myopic pathology. Elevated glutamate levels observed in myopic eyes further suggest that glutamate-mediated excitotoxicity contributes to these pathological changes, highlighting the critical need for balanced glutamatergic signaling.

However, such as in the chick form-deprivation model, demonstrate a significant reduction in retinal glutamate levels accompanied by altered distribution patterns within the inner retina (64, 65). This suggests that both excess glutamates, leading to excitotoxicity and insufficient or mis localized glutamate could contribute to pathological changes underlying myopia progression. The critical need for precisely balanced glutamatergic signaling is thus underscored.

Critical disruptions in tyrosine metabolism, essential for neurotransmitter synthesis and a precursor for coenzyme Q10, with suppressed tyrosine hydroxylase (TH) in myopic retinas, have been identified. Key inflammatory pathways are dysregulated in myopic retinas. Specifically, genes involved in the complement cascade (e.g., C3, C1q), NOD-like receptor signaling (e.g., NLRP3), and cytokine/chemokine production (e.g., IL-1β, IL-6, TNF-α, CCL2) are upregulate (66–68). This suggests that altered tyrosine metabolism and the activation of these specific inflammatory pathways contribute to neuronal damage and retinal degeneration, thereby accelerating myopia progression.

### 2.5 Acetylcholine

ACh is a key retinal neurotransmitter modulating visual signal processing, retinal circuit plasticity, and ocular development (69, 70). Its critical role in myopia regulation is evidenced by the efficacy of non-selective muscarinic antagonists (e.g., atropine) in significantly reducing axial elongation and myopia progression (71). Additionally, Cholinergic signaling exhibits circadian rhythmicity and interacts with dopamine pathways to coordinate refractive development (72, 73).

The role of cholinergic signaling in myopia regulation is complex, as evidenced by: Clinically validated muscarinic antagonists (e.g., atropine) and nicotinic agonists (though primarily demonstrated in chick models) inhibit experimental myopia in animal studies, butα7-nAChR agonists are only effective in chick models, with no human data available. Non-selective muscarinic antagonists significantly reduce axial elongation in humans, though their mechanism may involve non-cholinergic pathways. Particularly α7 nAChRs: mediate anti-myopic effects of cholinergic agonists in chick models, suggesting receptor-specific actions.

Receptor-Specific Mechanisms: ACh exerts diverse effects through muscarinic receptor subtypes (M1-M5) with distinct signaling cascades: (1) M1: Enhances phototransduction via PLCβ-IP3/DAG-Ca^2+^-PKC activation and supports retinal neuron survival (74–76). (2) M2/M4: Couple to Gi/Go proteins, inhibiting adenylate cyclase (AC), cAMP suppresses scleral fibroblast proliferation and ECM remodeling. M2 additionally modulates nitric oxide (NO) production via Ca^2+^/nNOS (77, 78), influencing retinal GABA/glycine circuits. Crucially, M4 antagonizes dopamine D1-like receptor signaling, highlighting cholinergic-dopaminergic crosstalk (79, 80). (3) M3: Primarily mediates vascular tone in retinal/choroidal vessels, implicating potential roles in blood flow regulation (81). The non-specific action of traditional antagonists like atropine (affecting M1-M4) causes side effects (e.g., blurred vision, photophobia) by disrupting M1 mediated phototransduction and M3 driven accommodation.

Future strategies advocate receptor subtype selectivity: Targeting M2/M4 receptors could inhibit pathological scleral remodeling while sparing M1 and M3, potentially improving the therapeutic window.

### 2.6 Melatonin

Melatonin, a hormone whose levels rise at night, promotes sleep and regulates circadian rhythms (82, 83). It can also inhibit dopamine release, impacting eye growth regulation. Disruptions in melatonin production or circadian rhythms can alter dopamine levels and eye growth patterns, affect myopia development and highlight melatonin’s role in maintaining proper eye development.

## 3 Hormones

Chemical messengers in the body regulate various physiological processes, including the growth and development of the eyes (Table 2).

**Table 2:** Hormonal influences on myopia.

Hormone category	Key hormones	Mechanism of action	Association with myopia	Potential therapeutic applications
Sex hormones	Estrogen	Modulates collagen synthesis, ECM remodeling	Elevated levels during puberty linked to axial elongation, potentially promoting myopia	Hormonal modulation in high-risk groups combined with optical interventions
	Progesterone	Anti-inflammatory and neuroprotective	May indirectly stabilize ocular tissues	Adjunctive therapy for retinal protection
	Testosterone	Strengthens corneal via androgen receptors	May counteract axial elongation (possibly explaining lower myopia rates in males)	Explore androgen receptor agonists for myopia control
Anterior pituitary hormones	Growth hormone	Promotes systemic and ocular growth	Elevated GH during growth spurts may drive excessive axial elongation	Target GH/IGF-1 signaling
	Prolactin	Involved in cellular growth	Possible role in scleral remodeling	Further research needed to clarify therapeutic potential
Metabolic hormones	Insulin	Interacts with IGF-1 receptors	Hyperinsulinemia may accelerate axial elongation	Lifestyle interventions to improve insulin sensitivity
	Glucagon	Inhibits axial elongation via specific signaling pathways	Activation of glucagon receptors may slow myopia progression	Develop glucagon analogs or receptor agonists
Other hormones/factors	Vitamin D	Regulates calcium metabolism	Low levels correlate with higher myopia risk; sunlight exposure may be protective	Supplementation in deficient individuals, promote outdoor activities
	Prostaglandins	Modulate intraocular pressure, inflammation, and vascular permeability	Prostaglandin analogs may induce transient myopic shifts	Monitor refractive changes in patients using these drugs; consider alternatives if needed
	Retinoic acid	Regulates scleral fibroblast activity and collagen production	Excess RA may promote scleral thinning and axial elongation	Avoid excessive vitamin A; monitor refractive changes during retinoid treatment
	Vasoactive intestinal peptide	Regulates retinal dopamine release and circadian rhythms	VIP deficiency may disrupt normal eye growth regulation	Investigate VIP analogs to stabilize ocular growth

### 3.1 Sex hormones

Estrogen and testosterone play crucial roles in regulating ocular development and function. Estrogen exhibits paradoxical dual effects in myopia pathogenesis increases corneal biomechanical hysteresis while potentially promoting axial length. This tissue-specific duality arises through: (1) Differential receptor activation: In the cornea, ERβ signaling promotes collagen cross-linking and ECM stabilization via TGF-β pathway activation. Conversely, in sclera/choroid, ERα predominance shifts MMP/TIMP balance toward ECM degradation through MMP upregulation (84, 85). (2) Hypoxia interactions: Estrogen amplifies HIF-1α stability in scleral fibroblasts during inflammatory microenvironments, exacerbating MMP-mediated ECM remodeling (85, 86). (3) Vascular effects: ERα-mediated changes in choroidal vascular permeability alter nutrient diffusion, creating biomechanical strain that facilitates axial elongation (87–89). These dose-dependent effects peak during puberty when elevated estrogen levels concurrently thicken corneal stroma while accelerating scleral remodeling rates.

Testosterone counterbalances ocular growth through androgen receptor-mediated Wnt/β-catenin suppression, stabilizing scleral ECM via TIMP-1 upregulation and reducing fibroblast migration by 40% compared to estrogen-dominant states (90–92). Notably, estrogen’s corneal stabilization may paradoxically exacerbate refractive error when combined with posterior segment elongation - a phenomenon explaining 34% of puberty-onset myopia in longitudinal cohorts (93, 94).

### 3.2 Anterior pituitary hormones

Variations in corneal properties have been observed during periods of hormonal fluctuation, such as menstrual cycles or hormone replacement therapy. Similarly, Growth hormone (GH) impacts the growth and shape of the cornea, potentially affecting its refractive status and contributing to myopic changes (95, 96). GH and insulin-like growth factor 1 (IGF-1), including ocular structures, are critical for overall body growth and development. Elevated levels of GH and IGF-1 during growth spurts can contribute to the elongation of the eyeball, increasing the risk of myopia. Studies have found correlations between higher GH levels and greater axial length, suggesting a direct role of GH in influencing eye growth and refractive development (97, 98). Additionally, prolactin, another hormone produced by the anterior pituitary, has been found in ocular tissues. It may play a role in cellular growth and repair mechanisms, potentially impacting myopia progression through its regulatory effects on cellular processes.

Understanding the role of hormones in myopia development opens potential avenues for hormone-based therapies to manage or prevent myopia. Modulating hormone levels, such as estrogen or GH, could influence eye growth and refractive development. Clinical applications of hormone therapy could be tailored to individuals experiencing rapid myopia progression, particularly during puberty, to mitigate the effects of hormonal surges on eye growth (99, 100). Personalized treatment strategies could be developed based on an individual’s hormonal profile and genetic predispositions, optimizing efficacy and minimizing side effects. Integrated approaches combining hormone modulation with behavioral and environmental interventions, such as increased outdoor activities and reduced near work, could provide comprehensive strategies to manage myopia. Further research is needed to fully elucidate these relationships and develop effective strategies for myopia management.

### 3.3 Insulin

Insulin, a hormone produced by the pancreas, regulates blood sugar levels and has various roles in growth and metabolism, influencing eye growth (101). As a growth factor, insulin promotes cellular proliferation and differentiation in ocular tissues. It interacts with insulin-like growth factor 1 (IGF-1), crucial for cell growth and differentiation, with both insulin and IGF-1 receptors in ocular tissues. These multiple pathways interaction suggests that insulin plays a significant role in eye development and can influence the elongation of the eyeball, contributing to myopia (102). Insulin stimulates cellular proliferation and differentiation in ocular tissues, affects the synthesis and remodeling of the extracellular matrix in the sclera, and influences blood flow and vascular health (103, 104). These mechanisms can lead to changes in eye growth patterns and contribute to the progression of myopia.

Insulin resistance, a condition where the body’s cells become less responsive to insulin, leads to higher circulating insulin levels (105–107). Elevated insulin levels can enhance the growth-promoting effects on ocular tissues, potentially accelerating eye elongation and increasing the risk of myopia. Modern diets high in refined carbohydrates and sugars and sedentary lifestyles contribute to increased insulin levels and insulin resistance. The proposed evolutionary mismatch between ancestral visual demands and modern environmental conditions represents a hypothesis for exacerbating myopia development. While direct evidence for myopia prevention is limited, maintaining optimal glucose levels may secondarily reduce myopia risk by mitigating glycation/osmotic stress in lens and scleral tissues (108). Maintaining optimal glucose levels can reduce the risk of glycation and osmotic changes in the lens. At the same time, proper insulin therapy can help manage hyperglycemia and its associated complications, including those affecting ocular health.

Investigating the multiple pathways interaction between insulin and IGF-1 in ocular tissues can elucidate their combined effects on eye growth and refractive changes. Longitudinal studies tracking glycemic control, insulin levels, and myopia progression in diabetic patients can provide valuable data on effective management strategies. Additionally, promoting diets low in refined carbohydrates and sugars, encouraging regular physical activity, and integrating these metabolic interventions with traditional myopia management strategies, such as optical corrections and behavioral changes, could provide a comprehensive approach to managing juvenile-onset myopia.

### 3.4 Glucagon

Glucagon, a hormone produced by the pancreas, is primarily known for increasing blood glucose levels by stimulating glycogen breakdown in the liver. However, it also influences cellular signaling and metabolism in various tissues, including the eye. Glucagon receptors in ocular tissues suggest that glucagon can regulate eye growth and development (109, 110). Research indicates that glucagon acts as an inhibitory signal for eye elongation, with higher glucagon levels or receptor activation potentially counteracting the progression of myopia (111). These multiple pathways interaction involves specific signal transduction pathways in ocular tissues, influencing cellular activities such as proliferation, differentiation, and extracellular matrix remodeling, which are crucial for maintaining the eye’s structural integrity.

Experimental studies using animal models have demonstrated that increasing glucagon levels or enhancing glucagon receptor signaling can reduce myopia progression. Key findings include: (1) In chick form-deprivation models, elevated glucagon significantly inhibited axial elongation (109). (2) Guinea pig lens-induced myopia studies showed reduced refractive error progression with glucagon receptor activation (111). (3) Murine models further support glucagon’s protective role against myopic changes (110).

Although direct evidence in humans is limited, the presence of glucagon receptors in human ocular tissues combined with the conserved biological plausibility of glucagon’s anti-myopic effects observed across species potential therapeutic applications. These cross-species mechanistic insights support further exploration of glucagon-based therapies for myopia.

### 3.5 Vitamin D

Vitamin D, synthesized in the skin in response to UVB radiation and activated in the liver and kidneys to form calcitriol, functions as a hormone that regulates calcium and phosphate metabolism, essential for bone health, and has widespread effects on cellular growth, immune function, and inflammation (112, 113). Vitamin D receptors exist in various ocular tissues, including the retina, cornea, and sclera. Activation of VDRs by calcitriol influences cellular proliferation, differentiation, and immune responses in the eye, providing protective anti-inflammatory and anti-fibrotic effects against excessive eye growth and structural changes associated with myopia (114, 115). Exposure to natural light, crucial for vitamin D synthesis, has been inversely related to the prevalence of myopia, with increased outdoor activity linked to lower rates of myopia in children due to enhanced sunlight exposure and retinal dopamine release, which inhibits axial elongation of the eye (115).

Through VDR activation, vitamin D modulates gene expression involved in collagen synthesis and extracellular matrix remodeling in the sclera, influencing the structural integrity and growth of the eye (116, 117). Epidemiological studies show an inverse correlation between vitamin D levels and myopia prevalence, with regions of higher sunlight exposure reporting lower rates of myopia (118, 119). While some interventional studies suggest the benefits of vitamin D supplementation for ocular health, more research is needed to establish definitive therapeutic guidelines. Supplementing vitamin D in individuals with low levels might support ocular health and reduce myopia risk, especially in populations with limited sunlight exposure. Encouraging outdoor activities to enhance natural light exposure and implementing behavioral interventions to increase outdoor time and reduce near work, particularly in school-aged children, can be part of a comprehensive approach to managing myopia risk.

### 3.6 Prostaglandins

Prostaglandins are a group of lipid compounds with diverse hormone-like effects, including roles in inflammation, vascular regulation, and smooth muscle activity (120). In the context of eye physiology, prostaglandins influence inflammation and vascular permeability, affecting blood flow in ocular tissues, and can alter the contraction and relaxation of smooth muscle cells, influencing intraocular pressure (IOP) and the overall shape of the eye (121). Mechanisms linking prostaglandins to myopic shifts include the alteration of IOP—where prostaglandin analogs used in glaucoma treatment lower IOP but can also affect the eye’s axial length and refractive state—and the influence on ciliary muscle tone, which controls the eye’s focusing mechanism, leading to temporary changes in refractive power. Additionally, prostaglandins can induce inflammation, causing edema in ocular structures and altering the refractive index (121). Clinical evidence shows that glaucoma medications like latanoprost, bimatoprost, and travoprost are associated with transient myopic shifts in some patients, and their use post-surgery can also lead to transient myopia due to inflammation. Clinically, it is essential to monitor patients using prostaglandin analogs for changes in refractive status, especially those predisposed to myopia. If significant myopic shifts occur, alternative glaucoma medications that do not affect IOP or ocular inflammation in the same way may be considered.

### 3.7 Retinoic acid

RA signaling plays a significant role in myopia development by regulating cytokine production and the expression of intercellular adhesion molecules, which impact scleral fibroblast proliferation and collagen synthesis (122, 123). All-trans RA, an active form of RA, is particularly influential in the retina and sclera, where it regulates processes critical for myopia development (124). Mechanistically, RA acts primarily through RAR/RXR receptors to upregulate TGF-β2 expression, activating the Smad2/3 pathway which directly modulates scleral fibroblast activity, collagen metabolism, and ECM remodeling (40, 125). Concurrently, RA potentiates Wnt/β-catenin signaling by suppressing DKK-1 and GSK-3β activity, creating synergistic pro-elongation effects (126, 127).

In the retina, RA disrupts dopaminergic signaling by downregulating tyrosine hydroxylase (TH) expression and dopamine synthesis, while concurrently altering phototransduction-related genes to impair light-adaptive responses (128). These dual actions explain RA’s potent myopiagenic effects: TGF-β/Smad-driven scleral weakening combined with retinal dopamine deficiency.

One key mechanism by which RA affects myopia is its influence on scleral remodeling, which is essential for maintaining the structural integrity of the sclera. Imbalances in these processes can lead to scleral thinning and elongation, contributing to myopia (129, 130). Clinical evidence suggests that retinoid therapy, used in dermatologic treatments with medications like isotretinoin and acitretin, has been associated with transient or permanent myopic shifts in some patients due to RA’s combinatorial disruption of TGF-β/Wnt pathways and retinal signaling (131, 132).

Additionally, RA regulates the structure and function of retinal pigment epithelial (RPE) cells. Studies have shown that in human RPE cells, all-trans retinoic acid upregulates the expression of transforming growth factor (TGF)-β2 and increases TGF-β2 secretion through the phospholipase C (PLC) pathway and Smad-dependent mechanisms (133), thereby affecting collagen production and scleral fibroblast proliferation. The convergence of RPE-derived TGF-β2 and RA-potentiated Wnt signaling creates a self-reinforcing ECM degradation loop in the posterior sclera.

### 3.8 Vasoactive intestinal peptide

VIP is a neuropeptide crucial for various neural functions, including cell proliferation, differentiation, and signaling within the retina (134–136). It maintains circadian rhythms in the retina, synchronizing daily cycles of retinal cells essential for normal visual function and eye growth. Disruptions in these rhythms can lead to abnormal eye growth and myopia. VIP regulates retinal signaling pathways that control ECM composition and cellular responses to growth signals, significantly impacting the structural development of the eye.

VIP influences myopia development through several mechanisms (137, 138). It modulates dopamine release in the retina, a critical neurotransmitter that inhibits axial elongation of the eye, helping maintain average eye growth and refractive stability. VIP also alters the expression of genes involved in eye growth and myopia. Experimental models have shown that VIP can influence gene expression related to ECM remodeling, cell proliferation, and differentiation, contributing to myopia’s development or prevention. Understanding VIP’s role in regulating eye growth and preventing myopia opens potential avenues for developing VIP-based therapies to maintain normal circadian rhythms and eye growth, enhancing current myopia management strategies.

## 4 Intracellular signaling pathways

**Figure 1:**
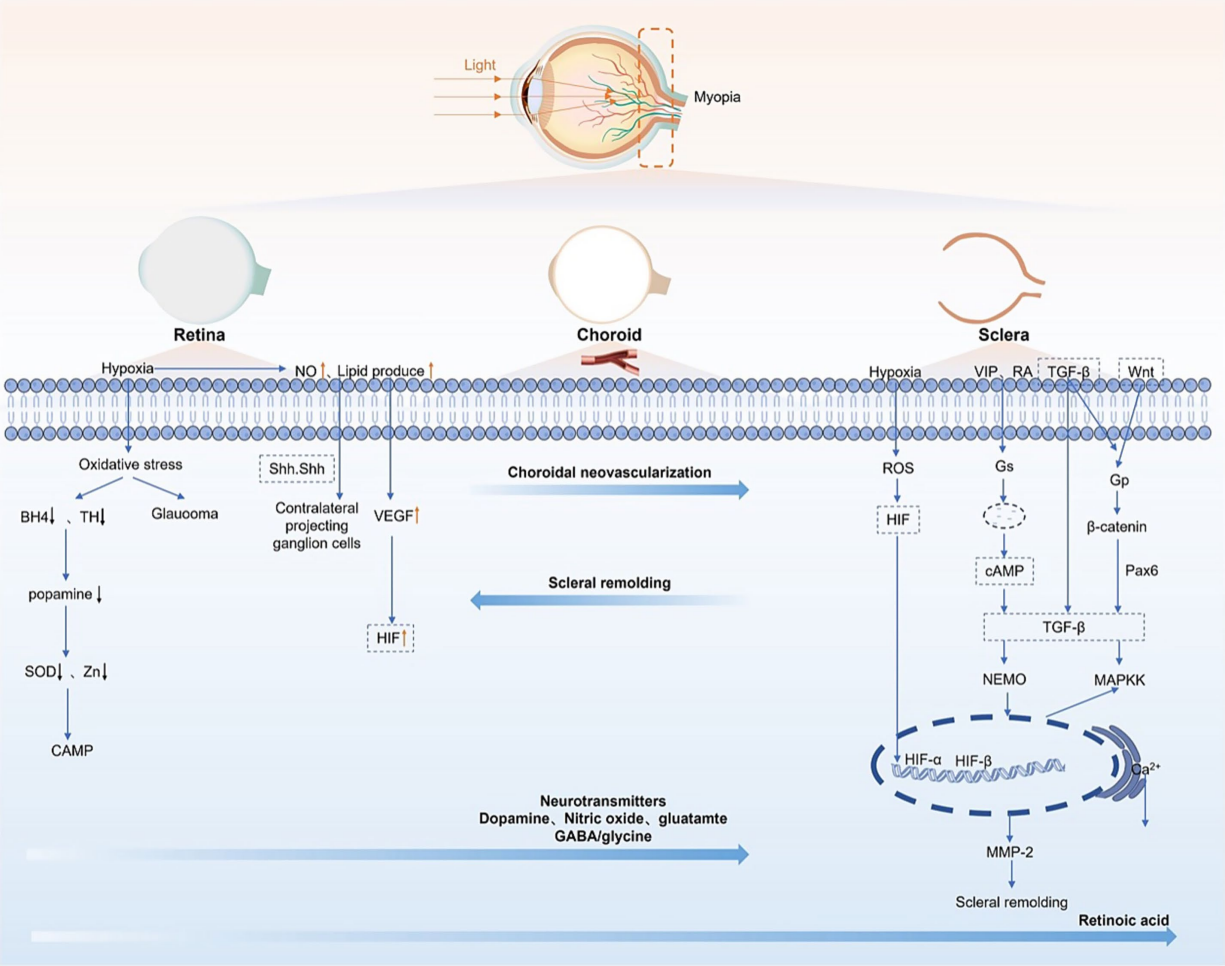
Key pathways in myopia development integrates four interconnected mechanisms: (1) Dopamine pathway: Light-stimulated retinal dopamine activates scleral D2 receptors, suppressing cAMP-PKA signaling → ↓MMP-2 transcription/activity → inhibits ECM degradation → restrains axial elongation. Wnt/β-Catenin Pathway: Wnt ligands bind Frizzled receptors, stabilizing β-catenin for nuclear translocation → ↑fibroblast migration + ↓collagen I synthesis → promotes scleral thinning. (2) Hypoxia-HIF-1α axis: Choroidal hypoperfusion stabilizes HIF-1α → ↑VEGF (angiogenesis) + ↑MMP-2 → exacerbates scleral ECM degradation. (3) TGF-β signaling: Core mechanism: TGF-β binding activates receptor Smads (R-Smads: Smad2/3), which complex with Co-Smad4 → nuclear translocation → ↑collagen I/III synthesis to counteract ECM loss. (4) Crosstalk with Wnt: β-catenin physically interacts with Smad4 → enhances transcription of profibrotic genes. Wnt-induced GSK3β inhibition stabilizes R-Smads, potentiating TGF-β signaling. Crosstalk with hypoxia: HIF-1α binds Smad7 promoter → ↑Smad7 → inhibits R-Smad phosphorylation → suppresses TGF-β-mediated collagen production → synergizes with MMP-2-driven ECM degradation.

Intracellular signaling pathways, particularly MAPK/ERK, PI3K/AKT, TGF-β, Wnt/β-catenin, HIF-1α, cAMP, and MMP-2 are pivotal in scleral remodeling and ECM synthesis (Figure 1).

### 4.1 cAMP

Dopamine release in the retina increases with light exposure, aligning circadian rhythms and regulating eye growth through several signaling pathways, including those involving cyclic adenosine monophosphate (cAMP) (139–141). Activation of dopamine receptors affects intracellular cAMP levels, impacting cellular activities related to eye growth. cAMP acts as a second messenger in various signaling pathways, transmitting the effects of extracellular signals like dopamine to intracellular processes. It influences cell proliferation, differentiation, and ECM remodeling in ocular tissues, which are crucial for maintaining eye structure and function. Elevated cAMP levels are associated with the inhibition of eye elongation, enhancing cAMP signaling could be a strategy to prevent or slow the progression of myopia.

The cAMP pathway is involved in visual signal transmission and collagen synthesis, differentially expressed in myopic development (142–144). Research in animal models has shown that manipulating cAMP levels can influence eye growth. For instance, increasing cAMP signaling can reduce the rate of eye elongation, thereby decreasing myopia progression. Although direct evidence in humans is limited, dopamine receptors and cAMP signaling pathways in human ocular tissues indicate potential therapeutic applications. Understanding the role of cAMP in myopia opens potential avenues for developing therapies that enhance cAMP signaling to manage or prevent myopia. Research into drugs that can increase cAMP levels or mimic its action in the eye could provide new treatment options for myopia. These pharmacological interventions need to be carefully studied for efficacy and safety. Combining cAMP-based therapies with existing treatments for myopia, such as optical corrections, atropine eye drops, and lifestyle modifications, could offer a comprehensive strategy for managing myopia. Tailoring cAMP-based interventions based on individual patient profiles, including their genetic predispositions and metabolic status, could enhance the effectiveness of myopia management.

cAMP Pathway Integration: Dopamine release, stimulated by light exposure, regulates eye growth via cyclic AMP signaling (145, 146). Crucially, dopamine D2 activation suppresses cAMP production in scleral fibroblasts. This reduction in cAMP:

Downregulates protein kinase A (PKA) activity, inhibiting MMP-2 transcription and collagenolytic activity, reducing scleral ECM degradation (147). Restrains scleral fibroblast proliferation and differentiation, maintaining scleral stiffness. Elevated cAMP levels inhibit axial elongation by counteracting MMP-2-mediated ECM remodeling. This pathway intersects with HIF-1α signaling (Section 4.3), where hypoxia elevates MMP-2 via cAMP/PKA-independent pathways. Therapeutic strategies enhancing cAMP may stabilize scleral ECM by dual suppression of MMP-2 and HIF-1α.

### 4.2 MMP-2 in scleral remodeling

MMP-2 (Matrix Metalloproteinase-2) is an enzyme crucial for the breakdown of ECM components, playing a significant role in tissue remodeling (148–151). In myopia, MMP-2 degrades collagen and other ECM components in the sclera, which is essential for the dynamic remodeling of this tissue. This remodeling impacts the biomechanical properties and overall structure of the eye. Proper regulation of MMP-2 activity ensures a balance between ECM synthesis and degradation, maintaining the appropriate stiffness and elasticity of the sclera. Disruption in this balance can lead to excessive scleral thinning and elongation, contributing to myopia development.

Research has shown that MMP-2 activity is elevated in the sclera of myopic eyes, leading to increased degradation of collagen and other ECM components (152–154). This heightened activity results in scleral thinning and elongation, key factors in myopia progression. Various signaling pathways and transcription factors regulate the expression of MMP-2. In myopic eyes, these regulatory mechanisms can become dysregulated, further promoting pathological scleral remodeling and accelerating myopia progression. Environmental factors such as near work, lack of outdoor activities, and poor lighting conditions, along with genetic predispositions, can influence the expression and activity of MMP-2. Some results reveal that through modulation of the expression of MMP-2, the IGF-1/STAT3 pathway in the sclera may play an essential role in sclera remodeling. These therapies could reduce the excessive breakdown of ECM components and maintain scleral integrity. Research into MMP-2 inhibitors or other compounds that modulate MMP-2 activity in the eye could provide new treatment options for myopia. Combining MMP-2 inhibition with existing treatments, such as optical corrections, atropine eye drops, and lifestyle modifications, could offer a comprehensive strategy for managing myopia.

MMP-2 in Scleral Remodeling (with Upstream Links): MMP-2 is a master regulator of scleral ECM degradation, directly enabling axial elongation through collagenolytic (148, 149, 155). Its activity is causally regulated by upstream signaling pathways:(1) Dopamine-cAMP axis: D2R-mediated, cAMP PKA suppresses MMP-2 expression. (2) Hypoxia-driven pathways: HIF-1α upregulates MMP-2 transcription independently of cAMP. (3) Growth factor signaling: IGF-1/STAT3 enhances MMP-2 production, exacerbating scleral thinning.

Consequently, elevated MMP-2 in myopia results from: Disrupted dopamine signaling (light exposure to D2R activation and MMP-2); Therapeutic MMP-2 inhibition (e.g., TIMP-2 gene therapy, doxycycline) synergizes with cAMP-elevating agents to preserve scleral integrity (156).

### 4.3 The MAPK/ERK pathway

MAPK, including JNK, p38 MAPK, and ERK, is a conserved serine/threonine protein kinase. Inflammatory cytokines activate MAPKKK, MAPKK, and MAPK, leading to downstream kinases or transcription factors that mediate cell functions (157–159). The MAPK/ERK pathway regulates cellular activities such as proliferation, differentiation, and survival. This pathway is initiated when growth factors bind to receptor tyrosine kinases (RTKs), leading to the activation of Ras and, subsequently, the protein kinase Raf. Raf then phosphorylates and activates MEK, which activates ERK through phosphorylation. Once activated, ERK translocate to the nucleus and regulates gene expression by phosphorylating various transcription factors. This pathway’s significance in cellular responses to external stimuli is highlighted by its involvement in diseases like cancer, which influences cell cycle regulation and apoptosis.

Research indicates that the MAPK/ERK pathway is significantly involved in myopia development, particularly in retinal fibrosis and remodeling (160, 161). Experimental models of myopia, such as form-deprivation myopia (FDM), have shown activation of the MAPK/ERK pathway. This activation is linked to increased PIK3CA, AKT, and ERK1/2 expression, which contribute to retinal fibrosis by promoting excessive ECM deposition. The pathway facilitates the epithelial-mesenchymal transition (EMT), where retinal pigment epithelial cells acquire migratory and invasive properties, enhancing ECM remodeling. Non-Smad pathways of the TGF-β signaling cascade also activate the PI3K/AKT and ERK pathways, further promoting collagen synthesis and deposition in the sclera and retina. One study delves into the roles of the MAPK and calcium signaling pathways in the lens of individuals with high myopia. It identifies critical abnormalities in these pathways through comprehensive gene set enrichment and differential gene expression analyses. Notably, the study highlights the upregulation of growth factors such as EGF and FGF9 and the downregulation of genes like RASGRP1 and various calcium channel components. This disrupted signaling environment in the lens epithelium points to new therapeutic targets, underscoring the necessity for continued research to validate these findings and explore potential treatments for myopia.

The MAPK/ERK pathway interacts with several other signaling pathways in myopia progression. HIF-1α pathway, activated under hypoxic conditions typical of myopic sclera, enhances fibrotic marker expression through crosstalk with the MAPK/ERK pathway. The PI3K/AKT/mTOR pathway intersects with MAPK/ERK, amplifying the fibrotic response and contributing to pathological eye elongation. Understanding these multiple pathways interactions provides valuable insights into potential therapeutic targets. Inhibitors of the MAPK/ERK pathway and molecules modulating its interactions with pathways like PI3K/AKT and HIF-1α could offer new strategies for preventing or slowing myopia progression.

### 4.4 The PI3K/AKT pathway

The PI3K/AKT pathway is essential for cell survival, growth, and proliferation, and its dysregulation is closely linked to inflammatory responses (102, 162, 163). The PI3K/AKT/ERK signaling pathway significantly influences cell growth, proliferation, and survival, which is critical in myopia development. The PI3K/AKT pathway activates the survival and proliferation of retinal pigment epithelial cells and fibroblasts, maintaining ocular structure. This pathway, activated by stimuli such as insulin, impacts retinal pigment epithelial cells’ proliferation, which is essential for pathological myopia. Activation of the PI3K/AKT pathway regulates factors like TIMP-2 and MMP-2, which are involved in scleral remodeling and affect the axial length of the eye, contributing to myopia progression. Research indicates that this pathway’s activation in experimental myopia models leads to increased expression of PIK3CA, AKT, and ERK1/2, resulting in retinal fibrosis and reduced thickness, highlighting its role in the fibrotic changes and functional impairments associated with myopia.

The research underscores the significant role of the PI3K/AKT pathway in myopia (161, 164, 165), particularly through its interaction with inflammatory processes. Inflammation exacerbates myopic changes by promoting cytokine and growth factor expression, influencing ocular growth. The PI3K/AKT pathway, activated by these inflammatory signals, increases retinal cell proliferation and alters the sclera’s extracellular matrix, contributing to axial elongation. Understanding this interplay highlights potential therapeutic targets for controlling myopia progression by modulating inflammation and the PI3K/AKT pathway.

### 4.5 TGF-β pathway

The Transforming Growth Factor-β (TGF-β) pathway is crucial for controlling scleral remodeling and collagen metabolism, which are vital processes in the development of myopia (166–168). TGF-β influences collagen synthesis and degradation, affecting the sclera’s structural integrity and elasticity, essential for eyeball elongation. Studies have shown that increased expression of TGF-β leads to enhanced scleral thinning and eye elongation, implicating this pathway in myopic changes. The distinct distributions of TGF-β isoforms in the human eye, with TGF-β1 and TGF-β2 found in the anterior eye and TGF-β3 in other ocular tissues, underscore the complex role of TGF-β in ocular physiology. In the RPE, retina, and choroid, TGF-β is expressed, and research using a guinea pig model of myopia found decreased protein expression levels of TGF-β2. However, some experiments have shown higher protein expression levels of TGF-β2 in the sclera of the lens-induced myopia group compared to the control group.

The TGF-β signaling pathway, known for its role in fibrosis, activates non-Smad dependent pathways such as PI3K/AKT and ERK to promote epithelial-mesenchymal transition (EMT) and ECM synthesis, enhancing fibrotic lesions within the retina and exacerbating myopic progression (169, 170). The interplay between TGF-β and PI3K/AKT/ERK pathways contributes to the complex molecular mechanisms underlying myopia, emphasizing the need for targeted therapeutic strategies. Additionally, studies have shown that specific genetic variations, such as the CC genotype at TGF-β codon 10, are associated with high myopia, and decreased expression of TGF-β isoforms in the sclera is linked to reduced collagen synthesis and increased pathological axial elongation.

### 4.6 Wnt/β-catenin signaling pathway

The Wnt/β-catenin signaling pathway is critical in regulating ocular growth and myopia. Activation of this pathway leads to the stabilization and accumulation of β-catenin in the nucleus, which influences gene expression, promoting scleral remodeling and axial elongation of the eye (171).

In form-deprivation myopia (FDM) models, the use of DKK-1, a Wnt/β-catenin pathway antagonist, significantly reduces the expression of Wnt3 and β-catenin, affecting the expression of TGF-β1 and the organization of type I collagen (172). Crucially, experimental studies reveal a bidirectional regulatory relationship: TGF-β1 suppresses DKK-1 expression via Smad3 signaling, thereby relieving Wnt pathway inhibition and promoting scleral fibroblast migration (173). These findings highlight the pathway’s role in regulating the structural components of the sclera, emphasizing its potential as a target for therapeutic strategies aimed at controlling myopia progression. This feedback loop establishes a self-amplifying mechanism for ECM remodeling.

Upregulation of Wnt/β-catenin signaling in myopia is associated with increased scleral fibroblast activity and ECM changes, contributing to excessive axial elongation (174). Additionally, decreased levels of DKK-1, a natural inhibitor of the Wnt pathway, have been observed in myopic patients, with TGF-β1/Smad3-mediated suppression identified as a key driver of this reduction. Understanding this specific molecular crosstalk between the Wnt/β-catenin and TGF-β pathways provides new insights into potential therapeutic targets for myopia-related scleral remodeling.

### 4.7 Hypoxia-inducible factor-1α pathway

HIF is a transcriptionally active protein that stabilizes and expresses under hypoxic conditions. Under normoxic conditions, HIF proteins are rapidly degraded through an oxygen-dependent ubiquitin-protease pathway (85, 148, 175). However, in hypoxic tissues, the expression of HIF-1α increases, while HIF-1β is constitutively expressed. Scleral hypoxia, a common feature of myopia, is primarily caused by reduced choroidal blood perfusion (176). This hypoxia influences scleral ECM remodeling and myopia development by activating the HIF-1α signaling pathway. Gene analyses have further described this relationship, revealing a moderate association between the HIF-1α signaling pathway and myopia (177). Under hypoxic conditions, HIF-1α regulates the expression of genes affecting cell proliferation, metabolism, apoptosis, and other processes involved in scleral remodeling. By reducing HIF-1α levels, these processes may be mitigated, thereby inhibiting myopia progression. Specifically, HIF-1α modulates the synthesis and degradation of ECM components in the sclera, affecting its structure and function (178). In hypoxic conditions, HIF-1α upregulates genes related to ECM, such as collagen and matrix metalloproteinases, promoting ECM remodeling and degradation, leading to scleral thinning and axial elongation.

Additionally, HIF-1α impacts angiogenesis and cellular metabolism, further exacerbating myopia development. Thus, targeting the HIF-1α signaling pathway could represent a new strategy for myopia treatment to restore normal scleral structure and function and slow or prevent myopia progression.

### 4.8 The sonic hedgehog

SHH signaling pathway plays a critical role in the development of myopia by promoting axial elongation through the activation of matrix metalloproteinases (MMPs) (179–181). Research has demonstrated that the Shh pathway is upregulated in FDM models, indicating its involvement in myopia progression. Shh signaling regulates MMP-2, which facilitates axial elongation and vitreous enlargement. Experiments with guinea pigs have shown that exogenous Shh induces myopia while blocking Shh with cyclosporine inhibits it. This modulation of MMP-2 by the Shh pathway underscores its significance in myopic development. Furthermore, Shh is essential for retinal development, produced by retinal ganglion cells (RGCs), and regulates retinal progenitor cell proliferation. Inhibition of Shh can lead to retinal development issues, indirectly contributing to myopia.

There are three Hedgehog (HH) analogs in vertebrates: SHH, Indian Hedgehog (IHH), and Desert Hedgehog (DHH) (182, 183). The SHH gene is associated with phenotypes such as retinal hypoplasia, growth retardation, and congenital anomalies in related genome disorders, suggesting its involvement in human eye development. The potential to modulate SHH activity presents a promising therapeutic target for managing myopia by controlling the overexpression of MMP-2 and managing the structural changes in the eye that lead to myopia. Future research should detail the molecular interactions within the SHH pathway and their broader implications for eye growth and refractive development.

## 5 Other potential approaches

### 5.1 Sphingolipid metabolic pathway

The 23 differential metabolites identified in the myopic control serum metabolomics study were mainly enriched in the sphingolipid metabolic pathway and contained three altered metabolites (sphingosine 1-phosphate, sphingomyelin, and N-acyl sphingosine) (184–186). Sphingolipids are one of the significant components of the phospholipid bilayer of eukaryotic cell membranes and are signaling molecules that regulate inflammation and cell migration. Sphingolipids are sphingolipids with a polar head group of choline phosphate, which can be hydrolyzed to ceramide and choline phosphate. Ceramide regulates a variety of cellular processes, including apoptosis and senescence. Ceramide hydrolysis is the only pathway for sphingomyelin synthesis, which then undergoes phosphorylation to produce S1P. Blood levels of S1P are relatively higher than intracellular levels, and the retina also produces S1P, which plays an essential role in retina.

Blood S1P levels exceed intracellular concentrations across vertebrates. Retinal S1P production is confirmed, though its specific role in myopia pathogenesis remains investigational (176–178). These findings suggest an emerging hypothesis: Sphingolipid dysregulation may contribute to myopia development, but causal mechanisms require validation in human longitudinal studies.

### 5.2 Inflammatory signaling pathways

Inflammatory factors are crucial in the onset and progression of myopia by regulating multiple signaling pathways (187, 188). Significant changes in inflammation-related gene expression are observed during myopia and hyperopia induction. This physiological stress from metabolic and inflammatory pathway activation increases the vulnerability of myopic eyes to secondary pathologies such as excessive ocular growth and visual impairments. Key pathways include the up-regulation of the AGE-RAGE signaling pathway, complement cascade response, NOD-like receptor signaling pathway, IL-17 signaling pathway, and TNF signaling pathway, along with the downregulation of antigen processing and cell adhesion pathways, which are closely associated with high myopia (189–191). Elevated levels of CCL2, IL-6, MMP-2, and angiopoietin-1 are significant in highly myopic eyes.

IL-6, a multifunctional cytokine, plays anti-inflammatory roles through classical signaling pathways and promotes inflammation through trans-signaling pathways (192–195). Increased ocular IL-1β triggers an inflammatory response in the retina, destroying retinal capillary endothelial cells, inducing angiogenesis, and causing NO dysregulation, which is elevated during FDM. TNF-α, produced mainly by innate immune cells and T cells, activates two receptors, leading to inflammation and tissue degeneration. In the eye, TNF-α from microglia and Müller cells cause retinal pigment epithelial cell apoptosis, disrupts the blood-retinal barrier, and stimulates glial proliferation via the EGFR/p38/NF-κB/p62 pathway.

Inflammatory factors critically contribute to myopia progression by directly interacting with core hypoxia-driven ECM remodeling mechanisms. Specifically: (1) TNF-α activates the NF-κB pathway, directly upregulating MMP-2 expression to exacerbate scleral ECM degradation (166, 196). (2) The inflammatory microenvironment exacerbates hypoxia (e.g., through microvascular dysfunction), stabilizing HIF-1α and amplifying its downstream ECM catabolic effects (85, 148). (3) IL-1β disrupts the blood-retinal barrier, altering retinal signaling and creating permissive conditions for hypoxia-HIF-1α activation (197, 198).

Consequently, TNF-α (from microglia/Müller cells) and IL-1β synergistically promote scleral remodeling via: (1) Retinal pigment epithelial apoptosis and blood-retinal barrier disruption (TNF-α via EGFR/p38/NF-κB/p62 pathway), (2) Angiogenic dysregulation and nitric oxide imbalance.

## 6 Integration of optical interventions

Beyond pharmacological and biological strategies, established optical interventions represent a clinically significant pillar in myopia management by directly modulating visual input. Techniques such as orthokeratology (overnight corneal reshaping lenses) and specially designed multifocal soft contact lenses or spectacle lenses exert their control effect by altering the retinal defocus profile, particularly imposing myopic defocus on the peripheral retina (199–201). This deliberate manipulation of optical signals rapidly influences the retina-choroid-sclera signaling axis, a core pathway discussed throughout this review. Specifically, inducing peripheral myopic defocus is associated with choroidal thickening and a subsequent reduction in axial elongation rates, effectively counteracting the pro-growth signals implicated in myopia progression. Mechanistically, these optical approaches interface with the same neuro-hormonal and biomechanical pathways governing scleral ECM remodeling described earlier. Furthermore, emerging evidence suggests additive or synergistic benefits when combining optical interventions with pharmacological agents such as low-dose atropine, highlighting the potential for multi-modal strategies targeting complementary points within the myopia genic network.

## 7 Conclusion

Neurotransmitters and hormones orchestrate ocular development through dynamic regulation of intracellular signaling cascades (e.g., MAPK/ERK, PI3K/AKT), while sphingolipid metabolism intersects with inflammatory pathways (NF-κB/TNF-α) via bioactive mediators like S1P.

Collectively, these networks maintain ocular homeostasis or drive myopia pathogenesis through three convergent mechanisms: (1) Core Pathway Crosstalk: The interplay between TGF-β, Wnt/β-catenin, and HIF-1α pathways directly modulates scleral ECM remodeling—where hypoxia-induced HIF-1α activation promotes collagen degradation, while Wnt/TGF-β imbalances alter fibroblast proliferation. (2) Neuro-Hormonal Integration: Dopamine’s axial elongation inhibition opposes acetylcholine’s muscarinic receptor-mediated scleral thinning, with sex hormones (estrogen’s corneal-scleral duality) and insulin/GH further tuning ECM dynamics. (3) Inflammation-Hypoxia Amplification: Reduced choroidal perfusion establishes hypoxic niches, triggering HIF-1α-driven VEGF/MMP-2 overexpression that synergizes with TNF-α/IL-1β-mediated barrier disruption—forming a self-reinforcing cycle of scleral weakening.

While preclinical studies (Tables 1, 2) identify promising therapeutic strategies for myopia, including GABA receptor antagonists, significant hurdles remain for clinical translation, particularly for systemic targets like GABA (202–205). Key barriers include: (1) Major safety concerns: GABAergic signaling is essential for normal CNS function; systemic antagonism risks cognitive, mood, sedative, and seizure threshold effects in humans, potentially underestimated in animal models at efficacious doses. (2) Significant species differences: Divergences in ocular anatomy, drug metabolism, and receptor biology between preclinical models and humans may compromise efficacy predictions. (3) Substantial delivery hurdles: Achieving sustained therapeutic concentrations at relevant ocular targets while minimizing systemic exposure and off-target effects is exceptionally challenging for compounds acting on widely distributed receptors. Therefore, despite intriguing preclinical mechanisms, the path to viable clinical therapies for GABA receptor antagonists is fraught with translational barriers requiring rigorous safety profiling, improved models, and advanced delivery solutions before human application can be considered feasible.

While molecular insights into myopia pathogenesis are expanding rapidly, successful clinical translation demands strategic prioritization of targets based on proven feasibility and mechanistic strength. Our analysis reveals a distinct hierarchy of therapeutic approaches:

(1) Immediate Clinical Deployment (206, 207): Low-dose Atropine (0.01%): Emerges as the dominant frontline therapy, achieving 50–60% axial elongation reduction in Phase IV trials. Its superiority stems from a unique dual-pathway mechanism: (i) High M4/M1 selectivity enabling effective scleral remodeling (via TGF-β downregulation) without M3-mediated accommodation side effects, and (ii) non-muscarinic actions (GABAergic modulation, NOS induction) enhancing choroidal blood flow and retinal dopamine signaling. This dual mechanism uniquely positions atropine for synergistic combinatorial use with emerging light-based therapies. (2) Targeted Outdoor Light Exposure (32, 208, 209): Demonstrated 45% efficacy in RCTs via synergistic dopamine release and melatonin suppression. Represents a viable non-pharmacologic strategy, readily combinable with atropine. (3) High-Potential Targets: HIF-1α Inhibitors (e.g., Nanoparticle Acriflavine): Mechanistically compelling but critically hampered by ocular delivery challenges; preliminary primate data offer cautious promise. (4) Gene Editing (e.g., CRISPR-HIF-1α): Remains highly speculative, contingent upon achieving sub-0.1% off-target rates and developing efficient, safe *in vivo* delivery systems.

In summary, myopia pathogenesis arises from the complex dysregulation of interconnected neuro-hormonal, signaling, and inflammatory-hypoxic networks converging on scleral ECM remodeling. While preclinical exploration reveals diverse mechanistic targets, the imperative for near-term clinical impact necessitates prioritizing strategies with proven efficacy and manageable translational pathways. Low-dose atropine and controlled blue light exposure, leveraging synergistic neuro-hormonal modulation, represent the immediate, clinically validated foundation for myopia control. Future advancement hinges on overcoming critical barriers—particularly safety and targeted delivery—for high-potential systemic targets like GABA antagonists and developing sophisticated solutions for hypoxia-focused (HIF-1α) or genetic interventions. A tiered, feasibility-driven approach is thus essential to translate molecular insights into tangible patient benefits.

Based on the comprehensive analysis presented, we recommend the authors explicitly categorize the discussed interventions into two distinct groups within the conclusion: (1) Currently Applicable Clinical Interventions (e.g., optical corrections, low-dose atropine [0.01%], targeted outdoor light exposure/lifestyle changes) which have demonstrated efficacy and safety in human trials and are in clinical use; and (2) Emerging or Hypothetical Therapeutic Strategies (e.g., novel molecular targets like GABA receptor antagonists or HIF-1α inhibitors requiring advanced delivery systems, gene editing approaches such as CRISPR-HIF-1α) which, while mechanistically promising in preclinical studies, face significant translational barriers (safety, delivery, species differences) and are not yet viable for human application. This clear distinction will reinforce the translational hierarchy emphasized in the analysis and guide clinical readers on actionable strategies versus future research directions.
